# Long-term outcomes of enzyme replacement therapy from a large cohort of Korean patients with mucopolysaccharidosis IVA (Morquio A syndrome)

**DOI:** 10.1016/j.ymgmr.2025.101189

**Published:** 2025-01-15

**Authors:** Juyoung Sung, Insung Kim, Minji Im, Yoon Ji Ahn, Sang-Mi Kim, Ja-Hyun Jang, Hyung-Doo Park, Tae Yeon Jeon, Kyung Rae Ko, Se-Jun Park, Jun Hwa Lee, Eun Young Kim, Chong Kun Cheon, Eungu Kang, Jung-Eun Moon, Young Bae Sohn, Hsiang-Yu Lin, Chih-Kuang Chuang, Shuan-Pei Lin, Sung Yoon Cho

**Affiliations:** a Department of Pediatrics, Samsung Medical Center, Sungkyunkwan University School of Medicine, Seoul, Republic of Korea; b Department of Laboratory Medicine and Genetics, Samsung Medical Center, Sungkyunkwan University School of Medicine, Seoul, Republic of Korea; c Department of Radiology, Samsung Medical Center, Sungkyunkwan University School of Medicine, Seoul, Republic of Korea; d Department of Orthopedic Surgery, Samsung Medical Center, Sungkyunkwan University School of Medicine, Seoul, Republic of Korea; e Department of Pediatrics, Samsung Changwon Hospital, Ajou University School of Medicine, Suwon, Republic of Korea; f Department of Pediatrics, Chosun University Hospital, Ajou University School of Medicine, Suwon, Republic of Korea; g Department of Pediatrics, Pusan National University Children's Hospital, Ajou University School of Medicine, Suwon, Republic of Korea; h Department of Pediatrics, Korea University Ansan Hospital, Ajou University School of Medicine, Suwon, Republic of Korea; i Department of Pediatrics, Kyungpook National University Hospital, Republic of Korea; j Department of Medical Genetics, Ajou University Hospital, Ajou University School of Medicine, Suwon, Republic of Korea; k Department of Pediatrics, MacKay Memorial Hospital, Taipei 10449, Taiwan; l Division of Genetics and Metabolism, Department of Medical Research, MacKay Memorial Hospital, Taipei 10449, Taiwan; m Department of Medicine, MacKay Medical College, New Taipei City 252, Taiwan

**Keywords:** Dysostosis multiplex, Elosulfase alfa, Morquio A syndrome, Enzyme replacement therapy, MPS IVA, Mucopolysaccharidosis

## Abstract

Mucopolysaccharidosis (MPS) IVA (Morquio A syndrome) is an autosomal recessive lysosomal storage disorder caused by a mutation affecting the enzyme *N*-acetylgalactosamine-6-sulfatase (EC 3.1.6.4, GALNS). Enzyme replacement therapy (ERT) has been shown to improve physical performance, quality of life, and respiratory function in patients with MPS IVA; however, owing to the rarity of MPS IVA, data on Korean patient characteristics are limited. This retrospective study reports clinical, radiographic, biochemical, and molecular findings, and analyzes long-term clinical outcomes, from the largest cohort of Korean patients with MPS IVA in a single center. The analysis included 17 patients from 14 families (58.8 % females; median [range] age at diagnosis 5.2 [1.8–33.7] years). The majority of patients (64.7 %) were classified as having a severe phenotype, 23 % had an intermediate phenotype, and 11.8 % had an attenuated phenotype. Skeletal manifestations and radiologic abnormalities at initial diagnosis included gait abnormality (35.3 %), short stature (23.5 %), chest deformity (23.5 %), scoliosis (17.6 %), kyphosis (11.8 %), dysmorphic face (6 %), hip pain (6 %), and leg deformity (6 %). Twelve different *GALNS* mutations were identified. Patients received ERT for a median (range) 7.4 years (3.0–12.1). Twelve patients reached final adult height, and all patients with the severe/intermediate phenotype had short stature (<3rd percentile). Hemiepiphysiodesis was the most common surgical intervention among patients with the severe/intermediate phenotype. Drug-related adverse events (urticaria, rash, and anaphylaxis) were reported in four patients but were managed with antihistamines or desensitization. At follow-up, patients experienced improvements in functional independence measure score, ejection fraction, and the 6-min walk test compared with the pre-treatment baseline. This study provides real-world evidence for long-term stabilization of functional independence, endurance, and respiratory function among patients **with MPS IVA** treated with ERT, with no new safety concerns identified.

## 1 Introduction

Mucopolysaccharidosis (MPS) IVA (Morquio A syndrome, OMIM #253000) is an autosomal recessive lysosomal storage disorder caused by a mutation in *GALNS*, which encodes the enzyme *N*-acetylgalactosamine-6-sulfatase (EC 3.1.6.4, GALNS) [1]. Deficiency of this enzyme leads to accumulation of keratan sulfate (KS) and chondroitin-6-sulfate, particularly in bone, cartilage, and their extracellular matrix [2]. MPS IVA is a rare condition with an estimated point prevalence ranging geographically from 1 per 323,000 to 1 per 1,872,000 [3,4]. Data from patients in Asia suggest a birth prevalence of 1 per 304,000 to 1 per 500,000 [3].

MPS IVA manifests mainly as short stature and progressive skeletal dysplasia, spinal cord compression, respiratory insufficiency, hearing loss, corneal clouding, and heart valvular disease; intelligence is unaffected [1]. There is wide variability in clinical presentation and disease severity, with effects extending to multiple other tissues and organs [5]. MPS IVA manifests within the first few years of life, leading to growth retardation and a wide range of progressive, long-term, irreversible physical impairments. If left untreated, symptoms may lead to early death, with a mean (standard deviation) of 25 (±17) years at age of death [6].

Enzyme replacement therapy (ERT) in the form of infusions of elosulfase alfa (Vimizim) was approved by the US Food and Drug Administration and European Medicines Agency in 2014 [2,7]. Although there is limited evidence to support positive effects of ERT on bone lesions and skeletal dysplasia, a meta-analysis has shown that ERT improves physical performance, quality of life (QOL), and respiratory function in patients with MPS IVA [8,9].

Owing to the rarity of MPS IVA, data on the disease characteristics of patients from Korea remain relatively limited. Clinical and genetic characteristics from six [10] and ten [11] patients with MPS IVA have been reported prior to the introduction of ERT. The MOR-004 (double-blind, randomized, placebo-controlled, 24-week, phase 3) trial, included seven patients from Korea [12,13]. In 2016, ERT was approved and available for treatment of patients with MPS IVA in Korea; in 2022, Lee et al. reported details of a further nine patients who received ERT [9]. Although the study of Lee et al. provided invaluable insights into the impact of ERT on a small group of patients over an average follow-up period of 3.7 years, there remains a lack of data on Korean patients with MPS IVA, especially in terms of longer-term outcomes.

In this retrospective study of patients with MPS IVA, clinical, radiographic, and biochemical findings and long-term clinical outcomes are analyzed from the largest cohort of patients in a single center in Korea.

## 2 Methods

### 2.1 Study design and population

This retrospective cohort study of patients with MPS IVA, as diagnosed by enzyme assays and molecular analysis, was conducted at the Samsung Medical Center, Seoul, a tertiary care center that treats rare diseases such as lysosomal storage disorders, including MPS. Clinical data were collected from electronic medical records from August 2003 to October 2023. This study was approved by the Institutional Review Board (IRB) of Samsung Medical Center (IRB number 2023–11-126) and conducted in accordance with the principles of the Declaration of Helsinki.

### 2.2 Treatment

Patients were treated with once-weekly infusions of elosulfase alfa 2.0 mg/kg, initiated as either part of the MOR-004 phase 3 clinical trial [13] or following approval of the treatment in Korea.

### 2.3 Analyses

Pre-treatment baseline measurements included medical history, results of physical examinations, skeletal survey, body gauge, radiographic findings, genetic data, echocardiogram, pulmonary function tests (forced expiratory volume in 1 s [FEV1] and forced vital capacity [FVC]), 6-min walk test (6MWT) [14], QOL [15] and pain assessment (Adolescent Pediatric Pain Tool survey) [16], eye examination (slit-lamp biomicroscopy, tonometry, refraction, and funduscopy), and functional independence measure (FIM) scores. In severely affected, non-ambulatory patients, we performed alternative tests such as grip strength testing, the Canadian Occupational Performance Measure [17], Adolescent Pediatric Pain Tool [16], and MPS-Health assessment questionnaire-52 items [18].

At diagnosis two different methods of reporting glycosaminoglycan (GAG)-derived disaccharides were assessed: normalization to urinary culture sensitivity (cetylpyridinium chloride [CPC] unit/g creatinine) and normalization to creatinine (as mg/mmol creatinine) [19]. Normalization to culture sensitivity yielded more consistent values than creatinine normalization. Urine KS was measured by liquid chromatography–mass spectroscopy/mass spectroscopy assay as previously reported by Martell et al. [20] and Oguma et al. [21]. At diagnosis GALNS mutations were assessed against the reference sequence NM 000512.4. Follow-up evaluations included echocardiogram, pulmonary function tests, 6MWT, urine KS, FIM scores, and QOL (Supplementary Table 1). Phenotypic disease was classified as either severe, intermediate, or attenuated according to the clinical course and radiologic findings [11]. Patients with growth retardation with a final adult height of less than 125 cm, and early symptoms (before 3.5 years of age) with typical skeletal changes were classified as severe; patients with a final adult height of more than 150 cm and only mild skeletal changes were classified as mild (attenuated); and patients in between were classified as intermediate [11]. Drug-related adverse events were recorded according to the treating physician's judgment.

### 2.4 Statistical analysis

Continuous variables are presented with descriptive statistics (i.e., either mean and standard deviation or median and range). Results of FIM, FEV1/FVC, and 6MWT at baseline and at last visit were compared with an paired *t*-test, where differences of *P*-value <0.05 were considered statistically significant. Results for the ejection fraction (EF) and urine KS, were found not to follow a normal distribution according to the Shapiro-Wilk test and were checked for statistical significance using the Wilcoxon signed rank test. Results were analyzed using Rex (3.7.0.0) and visualized using GraphPad Prism 10 (GraphPad Software, San Diego, CA, USA).

## 3 Results

### 3.1 Patients

The characteristics of 17 patients from 14 families who were included in the analysis (58.8 % [10/17] females; median [range] age at diagnosis, 5.2 [1.8–33.7] years) are listed in Table 1. Seven patients (41.2 %) started treatment as part of the MOR-004 trial, and the remaining ten patients (58.8 %) started after approval in Korea. Four patients (Patients 2, 4, 5, and 6) were diagnosed after the approval of elosulfase alfa and received this treatment directly. Six patients (Patients 1, 8, 13, 14, 16, 17) had been previously diagnosed and did not participate in the clinical trial and received elosulfase alfa following its approval. The majority of patients (64.7 %, 11/17) were classified as having a severe phenotype; 23.5 % (4/17) had an intermediate phenotype; 11.8 % (2/17) had an attenuated phenotype.

**Table 1:** Demographics, characteristics, biochemical, and genetic data of patients with MPS IVA.

Patient number		Phenotype	Sex	Initial presentation	Age at diagnosis (years)	GAG level (CPC unit/g creatinine) *(mg/mmol creatinine)	Urine KS (mcg/mL)	GALNS (pmol/min/mg protein) (nmol/17 h/mg protein)†	*GALNS* mutation^‡^
	1	Attenuated	M	Gait abnormality, hip pain	28.1	27.4	45.58	6.93†	*c.317 A >* *G [p.(Asn106Ser)]*, c.553delG [p.(Glu185Argfs*14)]
	2	Attenuated	M	Gait abnormality	9.3	166.3	113.7	4.71†	*c.281G* *>* *A [p.(Arg94His)]*, *c.1493C* *>* *T [p.(Pro498Leu)]*
	3	Intermediate	F	Scoliosis	10.3	195.4	10.54	0.3	*c.218 A >* *G [p.(Tyr73Cys)]*, *c.725C* *>* *G [p.(Ser242Cys)]*
Family 1	4	Intermediate	F	Gait abnormality	4.6	858.2	5.03	3.41†	*c.868G* *>* *A [p.(Gly290Ser)]*,?
	5	Intermediate	M	Leg deformity	5.2	23.8*	105.13	2.8†	*c.868G* *>* *A [p.(Gly290Ser)]*,?
	6	Intermediate	M	Chest deformity	33.7	2.2*	12.51	3.86†	*c.218 A >* *G [p.(Tyr73Cys)]*,?
	7	Severe	F	Short stature, kyphosis	5.2	585.2	109.53	3.1†	*c.451C* *>* *A [p.(Pro151Thr)]*, *c.752G* *>* *A [p.Arg251Gln)]*
	8	Severe	F	Chest deformity, gait abnormality	2.1	133.3	42.87	0.2	*c.451C* *>* *A [p.(Pro151Thr)]*, *c.1243-1G* *>* *A [p.(*?*)]*
Family 2	9	Severe	F	Kyphosis, scoliosis	4.3	192.7	116.97	<0.1	*c.451C* *>* *A [p.(Pro151Thr)]*, *c.1000C* *>* T [p.(Gln334*)]
	10	Severe	F	Chest deformity	1.8	472.5	187.3	0.3	*c.451C* *>* *A [p.(Pro151Thr)]*, *c.1000C* *>* T [p.(Gln334*)]
	11	Severe	F	Dysmorphic face	8.3	230.4	116.86	0.2	*c.1000C* *>* T [p.(Gln334*)], *c.1156C* *>* *T [p.(Arg386Cys)*
	12	Severe	F	Short stature	2.9	420	180.38	0.78	*c.1568 A >* G [p.(*523Trpext*92)], *c.1568 A >* G [p.(*523Trpext*92)] homo
Family 3	13	Severe	M	Gait abnormality	7	266.8	979.2	8.44†	*c.415G* *>* *A [p.(Gly139Ser)]*, *c.451C* *>* *A [p.(Pro151Thr)]*
	14	Severe	F	Chest deformity	5.3	358.8	287.4	6.53†	*c.415G* *>* *A [p.(Gly139Ser)]*, *c.451C* *>* *A [p.(Pro151Thr)]*
	15	Severe	M	Short stature, gait abnormality	7.3	167.5	116.9	1.46†	*c.853_855delTTC [p.(Phe285del)]*, *c.1000C* *>* T [p.(Gln334*)]
	16	Severe	F	Short stature	5.1	349	363.03	4.05†	*c.451C* *>* *A [p.(Pro151Thr)]*, *c.1462G* *>* *A [p.(Val488Met)]*
	17	Severe	M	Scoliosis	2.4	224.4	535.46	4.35†	*c.566* *+* *3 A >* T [p.(Trp141*)], *c.1019G* *>* *A [p.(Gly340Asp)]*

Reference ranges: GAG: ≤9 years old: <175 CPC unit/g creatinine; >9 years old: <85 CPC unit/g creatinine. * ≤9 years old: 5.2–16.7 mg/mmol creatinine; >9 years: 0–7.1 mg/mmol creatinine. Urine KS: <7.9 μg/mL. GALNS: 39–166 pmol/min/mg protein; 18–72 nmol/17 h/mg protein. ^‡^Reference sequence NM_000512.4

CPC, cetylpyridinium chloride; F, female; GAG, glycosaminoglycan; GALNS, *N*-acetylgalactosamine-6-sulfatase; KS, keratan sulfate; M, male.

At diagnosis GAG levels in patients ranged from 27.4 to 858.2 CPC unit/g creatinine (median 230.4 CPC unit/g) and exceeded 150 CPC unit/g creatinine in most patients with the severe phenotype (Table 1). The main symptoms or signs at the first hospital visit included gait abnormality (35.3 %, 6/17), short stature (23.5 %, 4/17), chest deformity (23.5 %, 4/17), scoliosis (17.6 %, 3/17), kyphosis (11.8 %, 2/17), dysmorphic face (5.9 %, 1/17), hip pain (5.9 %, 1/17), and leg deformity (5.9 %, 1/17). Urine KS was also elevated above the reference range of <7.9 μg/mL across the majority of patients (mean [standard deviation], 195.8 [244.6] mcg/mL).

Residual GALNS activity at diagnosis was below the relevant reference ranges for all patients. Twelve different *GALNS* mutations were identified. Notably, Patient 12 had a homozygous mutation of c.1568 A > G [p.(*523Trpext*92)]. The most common mutation was c.451C > A [p.(Pro151Thr)], affecting seven alleles, followed by c.1000C > T [p.(Gln334*)], affecting five alleles (Supplementary Fig. 1). None of the patients' parents were consanguineously married.

### 3.2 Clinical findings

Patients received ERT for a median (range) of 7.4 years (3–12.1; Table 2). A total of 12 patients reached their final adult heights during treatment. The median (interquartile range) final adult height in patients with the severe and intermediate phenotypes was 102.7 cm (102.2–131), and all these patients maintained short stature (<3rd percentile) at last follow-up. Three patients (Patients 8, 13, and 14), all with the severe phenotype, were non-ambulatory. Knock-knee was also common among patients with the severe and intermediate phenotype (93.3 %, 14/15). The majority of all patients experienced corneal opacity (82.3 %, 14/17), but none required corneal transplant surgery because their vision was preserved. Ten of the 17 patients had hearing loss (58.8 %, 10/17), with sensorineural and mixed type of sensorineural and conductive hearing loss in six and four patients, respectively. Five required hearing aid assistance. One patient had combined airway obstruction and restrictive lung disease, which necessitated respiratory support with positive pressure ventilation; no patients had cardiac problems requiring medication.

**Table 2:** History of treatment, height, and clinical findings, and surgical history of patients at last visit.

	Phenotype	Sex	Age at start of treatment	Duration of treatment (years)	H_i_ (cm)	H_i_ SDS	H_L_ (cm)	H_L_ SDS	Clinical findings	Surgical history
1	Attenuated	M	31.1	7.3	168.5	−1.06	168.5*	−1.1	G, CO	
2	Attenuated	M	10.1	6.9	133.7	−0.056	162.5	−1.4	G, GA, CO, DP	16.4 years old, kyphosis correction
3	Intermediate	F	17.2	12.1	141.6	0.557	142.2*	−3.4	SS, G, PC, GA, CO, AOM	18 years old pelvic osteotomy
4	Intermediate	F	4.7	5.7	100.8	−1.07	121.8	−2.2	SS, G, KK, GA	Hemiepiphysiodesis
5	Intermediate	M	5.3	3	105.1	−0.957	117.4	−1.6	SS, G, KK	
6	Intermediate	M	34.0	4.6	134.2	−7.4	137.2*	−6.6	SS, G, PC, KK, GA, CO, DP, AOM, HL	33.2 years old, total hip replacement arthroplasty
7	Severe	F	12.3	12	95.7	−2.216	124.8*	−6.7	SS, G, PC, KK, GA, CO	Hemiepiphysiodesis
8	Severe	F	26.6	7.3	NA	NA	97*	−12.1	SS, G, PC, KK, GA, CF, CO, RP, DP, AOM, HL	
9	Severe	F	9.6	12	94	−4.4	102.7*	−11.0	SS, G, PC, KK, GA, CF, CO, HL	1.8 years old, varus derotation osteotomy Hemiepiphysiodesis
10	Severe	F	5.0	12	84.8	0.35	100*	−11.5	SS, G, PC, KK, GA, CF, CO, HL	15.1 years old, C1 decompression Hemiepiphysiodesis
11	Severe	F	12.2	12.1	96.3	−4.869	101.7*	−11.2	SS, G, KK, GA, CF, CO	Hemiepiphysiodesis
12	Severe	F	5.3	12.1	87.2	−1.591	97.8*	−11.9	SS, G, PC, KK, GA, CF, AOM, HL	Hemiepiphysiodesis
13	Severe	M	23.5	7.3	103	−11.741	103*	−11.7	SS, G, PC, KK, GA, CF, CO, HL	
14	Severe	F	21.9	7.3	109.5	−9.712	109.5*	−9.7	SS, G, PC, KK, GA, CF, CO, HL	
15	Severe	M	8.3	12	89.9	−6.164	102.7*	−10.0	SS, G, PC, KK, CF, CO, AOM, HL	Hemiepiphysiodesis
16	Severe	F	8.4	7.4	94.7	−2.957	106	−10.1	SS, G, PC, KK, GA, CF, CO, DP, AOM, HL	Hemiepiphysiodesis
17	Severe	M	5.7	7.4	86.6	−−1.15	105.2	−5.9	SS, G, PC, KK, GA, CF, CO, DP, AOM, HL	Hemiepiphysiodesis

AOM, recurrent acute otitis media; CF, coarsening of facial features; CO, corneal opacity; DP, dental problem; F, female; G, gibbus; GA, gait abnormality; H_i_, initial height; H_L_, height at last visit; HL, hearing loss; KK, knock-knee; M, male; PC, protrusion of the chest; RP, respiratory problem; SDS, standard deviation score; SS, short stature.

### 3.3 Radiographic findings

**Fig. 1:**
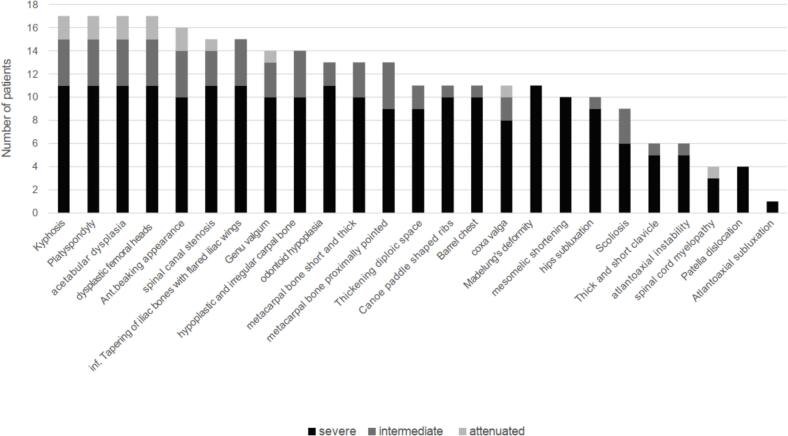
Summary of radiographic findings of patients with mucopolysaccharidosis IVA.

**Fig. 2:**
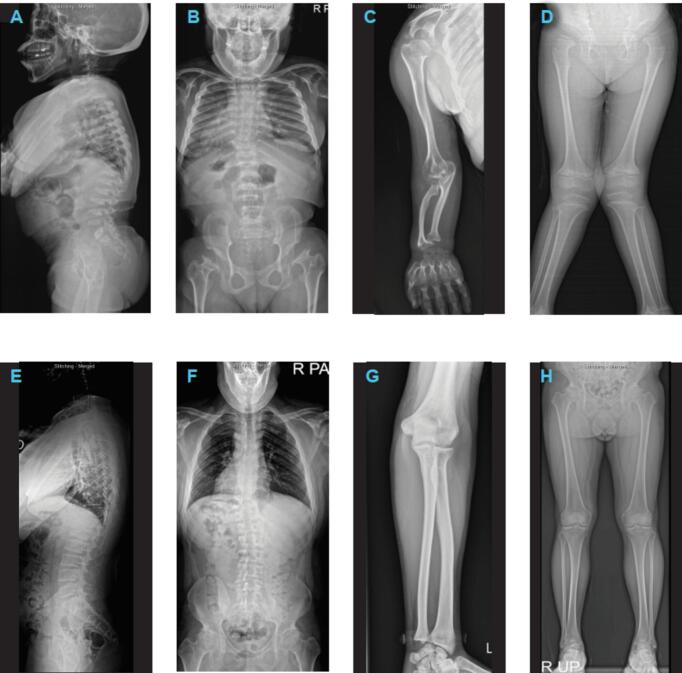
Radiographs of patient 12 (severe type) and patient 1 (attenuated). Patients 12: A, lateral (17.3 years); B, anterior (17.3 years); C, forearm (17.3 years), D, whole leg (6 years old prior to hemiephiphysiodesis). Patient 1: E, lateral (28.1 years), F, anterior (28.1 years); G, forearm (28.1 years); H, whole leg (28.1 years).

Notably, all patients (17/17), regardless of phenotype, developed kyphosis, platyspondyly, acetabular dysplasia, and dysplastic femoral heads (Fig. 1). Radiographs illustrating the deformities of the hip, spine, forearm, and legs of patients with severe and attenuated phenotypes are shown in Fig. 2A–D and Fig. 2E–F, respectively. With regard to surgical interventions, more than half of patients with severe or intermediate phenotype (60 %, 9/15) underwent hemiepiphysiodesis (Table 2). Patient 15 (severe phenotype) underwent three procedures between 7 and 20.3 years old and experienced progressive improvement in limb alignment, as illustrated in Supplementary Fig. 2. Three patients with severe or intermediate phenotype underwent procedures to correct hip dysplasia, including pelvic osteotomy (Patient 3, 18 years old), varus derotation osteotomy (Patients 9, 1.8 years old), and total hip replacement arthroplasty (Patient 6, 33.2 years old). Two patients underwent spinal surgeries, including C1 decompression (Patient 10, severe, 15.1 years old; Supplementary Fig. 3).

Four patients experienced drug-related adverse events (Supplementary Table 2). Two cases of urticaria and one case of rash were managed with antihistamines; one case of anaphylaxis was managed through desensitization; no adverse events led to discontinuation of ERT.

**Fig. 3:**
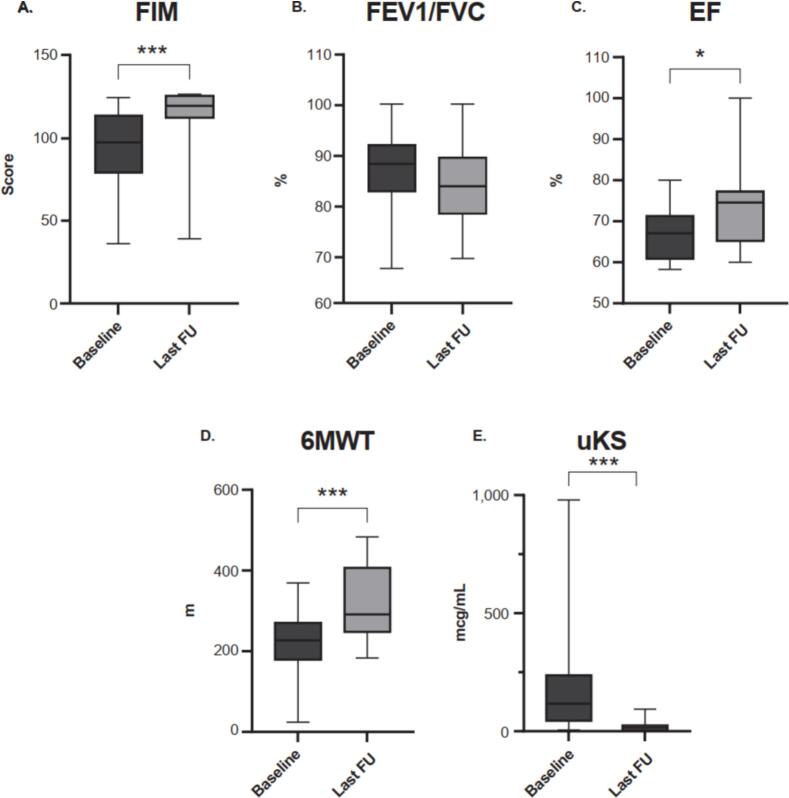
Changes in **A** FIM^†^, **B** FEV1/FVC^‡^, **C** EF^†^, **D** 6MWT^‡^, and **E** Urine KS^†^ from pre-treatment baseline to last visit. ^†^Number of patients tested = 17 (A, C, E), ^‡^Number of patients tested = 14 (B, D). 6MWT, 6-min walk test; EF, ejection fraction; FEV1, forced expiratory volume in 1 s; FIM, functional independence measure; FU, follow-up; FVC, forced vital capacity; KS keratan sulfate.

A comparison of pre-treatment baseline and follow-up examination results is shown in Fig. 3. All 17 patients underwent a comprehensive evaluation that include FIM, echocardiogram, and urine KS. Additionally, all but three non-ambulatory patients underwent pulmonary function tests and 6MWT. Results for FEV1/FVC appeared to be maintained across all 14 patients tested. Improvements of FIM score, ejection fraction, and 6MWT were statistically significant. Urine KS levels decreased sharply from baseline (Supplementary Fig. 4).

**Fig. 4:**
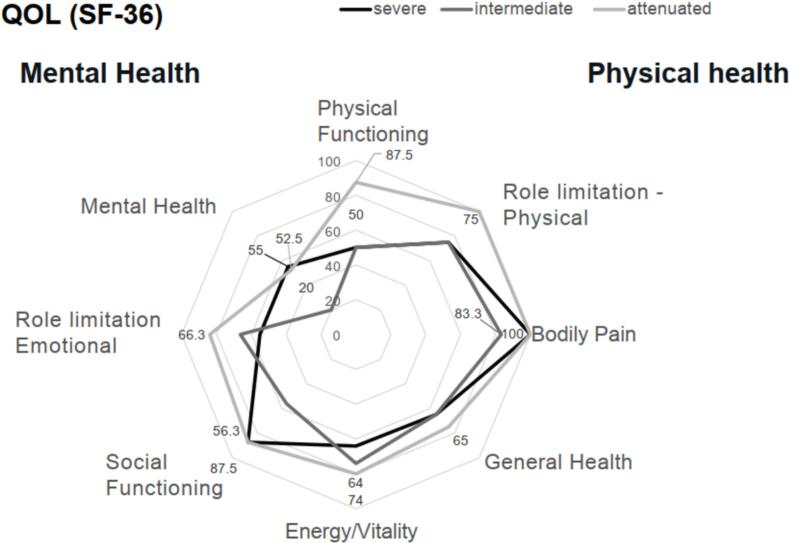
QOL assessment of patients at last visit. QOL, quality of life; SF-36, Short Form-36.

The results of QOL evaluations (Short Form-36) suggested that patients experienced a greater burden on their mental than their physical health (Fig. 4). Mean scores for mental health were consistently <60 among all phenotypes. Conversely, scores for bodily pain were > 80 among all phenotypes. Patients with the attenuated phenotype tended to report more favorable scores than patients with severe or intermediate phenotypes, particularly in the domains of physical functioning and role limitation (both physical and emotional). Scores for physical and mental health were strongly correlated (Supplementary Fig. 5).

## 4 Discussion

This is the largest study of the clinical course of patients with MPS IVA who received ERT in Korea. Patients with different phenotypes presented with a variety of related conditions sharing some common features. Symptoms of MPS IVA typically become apparent from around 2 years of age [22] and patients with MPS IVA have been recognized to present with diverse skeletal abnormalities, which necessitate consideration in differential diagnosis of other skeletal disorders such as multiple epiphyseal dysplasia, some forms of spondyloepiphyseal dysplasia, and bilateral Perthes-like disease [22]. The most common first symptom at initial presentation was gait abnormality.

The relatively high prevalence of the severe phenotype identified here is overall in line with the results of studies from other Asian populations [[23], [24], [25]]. Notably, among the current case series, the attenuated phenotype may be easily mistaken for other diseases when based on X-ray alone; this suggests that the disease may be underdiagnosed, as reflected by the small number of patients. Recent advances in genetic testing (such as next-generation sequencing) are expected to improve the chances of early detection of patients with the attenuated phenotype [22].

Owing to the heterogeneity of biochemical data among this small number of patients, it was not possible to identify correlations with phenotypical characteristics. Abnormalities of the spine (gibbus/kyphosis) and hips (platyspondyly, acetabular dysplasia, dysplastic femoral heads) were common among all patients in this study and in line with previous reports of patients with MPS IVA from other regions [26].

Patients in our study with the severe and intermediate phenotypes were all of shorter stature and tended to have abnormalities, including protrusion of the chest, gait abnormalities, and corneal opacity; three patients with the severe phenotype required the use of mobility devices. Neither of the two patients with the attenuated phenotype in this study had short stature or apparent knock-knee at diagnosis; however, one of them presented with genu valgum on later X-ray examination, and both patients had mild corneal opacities. All patients, regardless of phenotype, had X-ray findings of kyphosis, platyspondyly, acetabular dysplasia, and dysplastic femoral heads. Anterior beaking appearance, spinal canal stenosis, and genu valgum were also common features among patients of all phenotypes. These findings should be considered carefully, for example, when assessing a suspected case of MPS IVA, and may motivate definitive diagnosis by enzyme assays and molecular analysis.

Hemiepiphysiodesis was the most common surgical intervention among the patients and is recommended to be implemented as early as possible during growth in any patients with signs of genu valgum [27]. Appropriately timed orthopedic surgery is important to restore limb alignment, particularly in patients with the severe phenotype, who tend to experience greater deterioration of mobility. Annual spinal magnetic resonance imaging is also recommended for all pediatric patients, to assess cervical myelopathy and the need for decompression surgery. Key risks for cardiorespiratory failure and neurological complications associated with skeletal manifestations among patients with MPS IVA particularly motivate the need for multidisciplinary care from primary care physicians, pulmonologists, cardiologists, and orthopedic surgeons [6].

Notably, the genetic variants were heterogeneous among these patients. The most common variant was c.451C > A [p.(Pro151Thr)] (*n* = 7, all severe types) among 31 alleles, which has been reported in the Korean and Chinese literature [5,10]. The enzyme activity of patients with this mutation was low, ranging from <0.1–0.3 pmol/min/mg protein and 3.1–8.44 nmol/17 h/mg protein, and are thought to be associated with the severe phenotype. In addition, variant c.1000C > T [p.(Gln334*)] was found only in the severe type, while variant c.218 A > G [p.(Tyr73Cys)] and c.868G > A [p.(Gly290Ser)] were found in the intermediate type. The genotype-phenotype correlation analysis is limited because only one patient (Patient 12) has the homozygous mutation, and further research is needed.

All patients were diagnosed by polymerase chain reaction (PCR) and direct sequencing of the *GALNS* gene, except for patient 1, who underwent a next-generation sequencing (NGS) panel for skeletal dysplasia disorders. However, only one variant was identified in three patients, as reported in the literature that 10.7 % of MPS IVA patients have only one identified allele [10]. Considering the potential for hidden variants, additional molecular diagnostic tests are planned to find deep intronic variants, large deletions, or gross rearrangements. The primer sequences should also be reviewed to check allele dropout events caused by a common benign variant in the primer binding site [28].

All patients who underwent tests in this study showed improvements in FIM score, ejection fraction, 6MWT, and urine KS. The 6MWT distance increased by a median of 33 % (range 11–82 %) when comparing pre-treatment to last follow-up. The minimal important difference (MCID) for MPS IVA syndrome remains to be established; however, the MCID for other chronic diseases is 7–9 % [14]. Considering the 14.9 % improvement with elosulfase alfa in the phase 3 clinical trial [12,14], the improvement observed in our patients is substantial. The analysis showed that all ambulatory patients had improvement in their 6MWT, suggesting that ERT is effective in maintaining functionality and endurance. These improvements are in line with results of clinical trials [12,29] and long-term observational studies [30] of patients treated with ERT.

Pulmonary function did not worsen, compared with pre-treatment baseline, while receiving ERT. However, given the overall decline in pulmonary function at long-term follow-up of approximately 10 years [31], compared with the positive results in the relatively short-term clinical trials [12], patients must be carefully monitored as they may require non-invasive ventilation, tonsillectomy, and tracheal reconstruction in the long term. For non-ambulatory patients who were unable to perform pulmonary function tests or the 6MWT, we used tests such as grip strength testing, the Canadian Occupational Performance Measure, Adolescent Pediatric Pain Tool, and MPS-Health assessment questionnaire-52 items to set individualized goals, which were then used to assess the effectiveness of ERT.

In the present study, the youngest patient started ERT at 5 years old; there remains a need for further studies of effectiveness in patients starting treatment before 1 year old [9]. However, in most patients, growth retardation and skeletal progression continued despite of ERT. Previous studies have demonstrated that ERT does not reduce vacuoles in chondrocytes or abnormal extracellular matrix due to poor penetration of enzyme into avascular tissue [32]. There have been no reports of efficacy of ERT in terms of final adult height or skeletal deformities [2]. To address these unmet needs, the development of bone-targeted therapies, such as substrate degradation enzyme therapy, gene therapy (adeno-associated virus or lentivirus), or pharmacological chaperone therapy, is necessary.

Although urine KS values rapidly decreased, the changes in values from pre-treatment baseline did not appear to correlate with the severity of progressive skeletal problems. This pattern may reflect the limitation of KS levels and the increasing chance of false negatives in older patients. Thus, alternative biochemical markers are needed for monitoring patients with ERT. Additionally, lifestyle tracker apps to review patients' all-day record of activity and sleep habits might be helpful. Four patients experienced drug-related adverse events, which were managed with antihistamines or desensitization, and all patients continued ERT without interruption.

In the QOL evaluations of the patients, the overall score tended to be lower among patients with the severe/intermediate phenotype than those with the attenuated type. Mental health scores were low across all phenotypes. There was a significant correlation between patients' perceived physical health and mental health. Patients with MPS IVA have severe short stature and skeletal deformities, but their intelligence is preserved, allowing them to better understand their disease course and feel a greater psychological burden. All of our patients with the severe type had severe short stature of less than 110 cm final adult height, and two patients received psychological counseling. Depression, social adjustment problems and anxiety are possible as a result of physical dwarfism, and self-esteem may be lowered if independent living is difficult. Their mental health problems should not be overlooked and they should be assessed regularly for quality of life and psychological symptoms. Patients should be able to receive psychosocial support and, if necessary, psychiatric intervention at the appropriate time.

Limitations of this study include the retrospective single-arm, single-center study design. Two sibling patients (Patients 13 and 14) have recently been lost to follow-up, owing to the difficulty of their visiting the hospital. Further studies are warranted with QOL instruments specific to the features of patients with MPS IVA.

## 5 Conclusions

This study represents the longest and largest observational study of patients with MPS IVA to date in Korea, reflecting over 12 years of experience in 17 patients. This study provides real-world evidence for long-term stabilization of functional independence, endurance, and cardiopulmonary function among ERT-treated patients, with no new safety concerns identified. Care for patients with MPS IVA should include regular follow-up evaluations and a multidisciplinary approach from specialists, including mental health professionals. Although ERT may improve physical performance, almost all patients showed progression of skeletal deformities, and orthopedic surgery was needed in more than half of cases. There are several unmet medical needs for skeletal abnormalities in patients with MPS IVA that need to be addressed through earlier diagnosis, new emerging treatment (e.g., targeted therapy, antioxidant and anti-inflammatory agents, and adeno-associated virus gene therapy), or more effective management.

## Funding

This work, related to skeletal dysplasia, was supported by a 10.13039/501100003725National Research Foundation of Korea (NRF) grant funded by the Korea government (MSIT) (NRF-2021R1G1A1092117). Medical writing was funded by BioMarin Pharmaceutical Inc. The funder was not involved in any aspect of the study design, data analysis or manuscript development.

## Ethics approval and consent to participate

The protocol was approved by independent ethics committees/institutional review boards (IRB number 2023–11-126), and all patients provided written informed consent prior to the conduct of any study-related procedures. The study was conducted in compliance with applicable laws and regulations, the International Conference on Harmonization Guidelines for Good Clinical Practice, and the Declaration of Helsinki.

## CRediT authorship contribution statement

**Juyoung Sung:** Writing – original draft. **Insung Kim:** Investigation. **Minji Im:** Conceptualization. **Yoon Ji Ahn:** Investigation. **Sang-Mi Kim:** Methodology. **Ja-Hyun Jang:** Validation. **Hyung-Doo Park:** Validation. **Tae Yeon Jeon:** Data curation. **Kyung Rae Ko:** Resources. **Se-Jun Park:** Resources. **Jun Hwa Lee:** Resources. **Eun Young Kim:** Resources. **Chong Kun Cheon:** Resources. **Eungu Kang:** Resources. **Jung-Eun Moon:** Resources. **Young Bae Sohn:** Resources. **Hsiang-Yu Lin:** Data curation. **Chih-Kuang Chuang:** Methodology. **Shuan-Pei Lin:** Supervision. **Sung Yoon Cho:** Writing – review & editing.

## Declaration of competing interest

The authors have no relevant financial relationships or conflicts of interest to disclose.

## Data Availability

The data that has been used is confidential.

## Appendix A Supplementary data

Supplementary material Image 1
