# Supplementary material for: Long-term outcomes of enzyme replacement therapy from a large cohort of Korean patients with mucopolysaccharidosis IVA (Morquio A syndrome)
Source: Mol Genet Metab Rep. 2025 Jan 15;42:101189. doi: 10.1016/j.ymgmr.2025.101189 (PMC11783393; doi:10.1016/j.ymgmr.2025.101189)
Supplement: Supplementary file 1 — Supplementary material [file mmc1.docx]

#### Supplementary Information

**Table 1.** Follow-up evaluation protocol

| **Pre-treatment** | **Biannual** | **Annual** | **Every 1–3 years** | **As clinically indicated** |
| --- | --- | --- | --- | --- |
| Medical history | Medical history | Abdomen US | Spine MRI | Sleep study |
| Physical examination | Physical examination | Echocardiogram | Eye examination | Bone densitometry |
| Neurologic examination | Neurologic examination | Pulmonary function test | Hearing test |  |
| Skeletal survey (X-ray) | Growth evaluation | QOL and pain assessment | Dental evaluation |  |
| Echocardiogram | Endurance test (6MWT) | Cervical spine imaging |  |  |
| Pulmonary function test | FIM score |  |  |  |
| Endurance test (6MWT) |  |  |  |  |
| FIM score |  |  |  |  |
| QOL and pain assessment |  |  |  |  |
| Eye examination |  |  |  |  |
| Hearing test |  |  |  |  |
| Dental evaluation |  |  |  |  |

6MWT, 6-minute walk test; FIM, functional independence measure; MRI, magnetic resonance imaging; QOL, quality of life; US ultrasound.

**Table 2.** Summary of drug-related adverse events

| **Drug-related adverse event** | **n (%)** | **Management** |
| --- | --- | --- |
| Urticaria | 2 (11.7%) | Antihistamine, antipyretics, prednisolone |
| Rash | 1 (5.8%) | Antihistamine |
| Anaphylaxis | 1 (5.8%) | Desensitization |


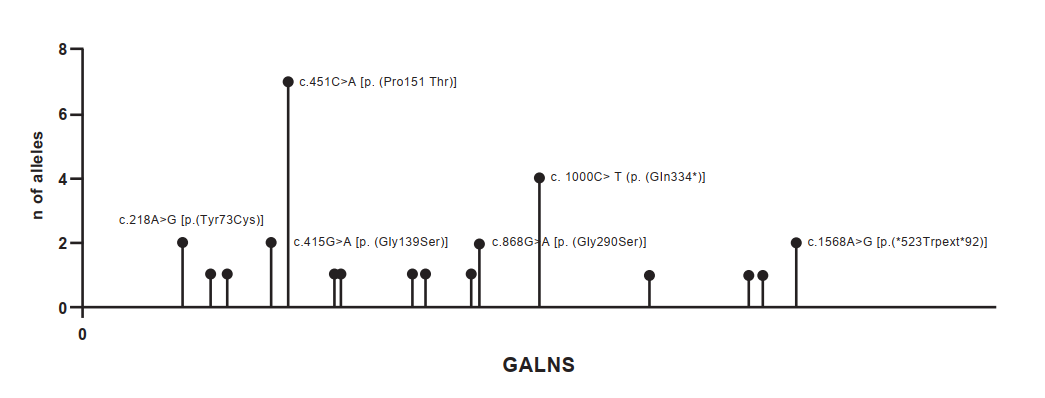


**Figure 1**. Schematic representation of mutations of the *GALNS* gene


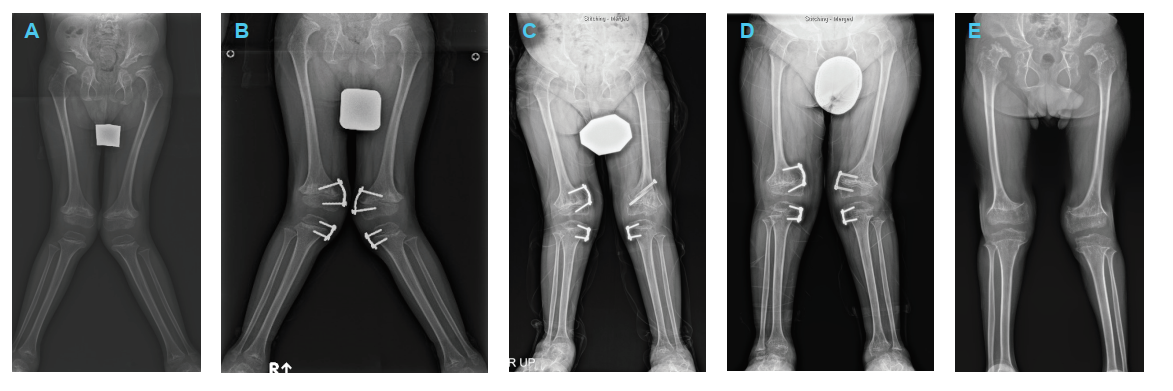


**Figure 2**. Radiographic findings of Patient 15 (severe): **A** Genu valgum, 7 years old; **B** 1^st^ operation hemiepiphysiodesis, 7.3 years old; **C** 2^nd^ operation femur osteotomy, hemiepiphysiodesis, 11.1 years old; **D** 3^rd^ operation hemiepiphysiodesis; 13.1 years old; **E** Follow-up at 20.3 years old.


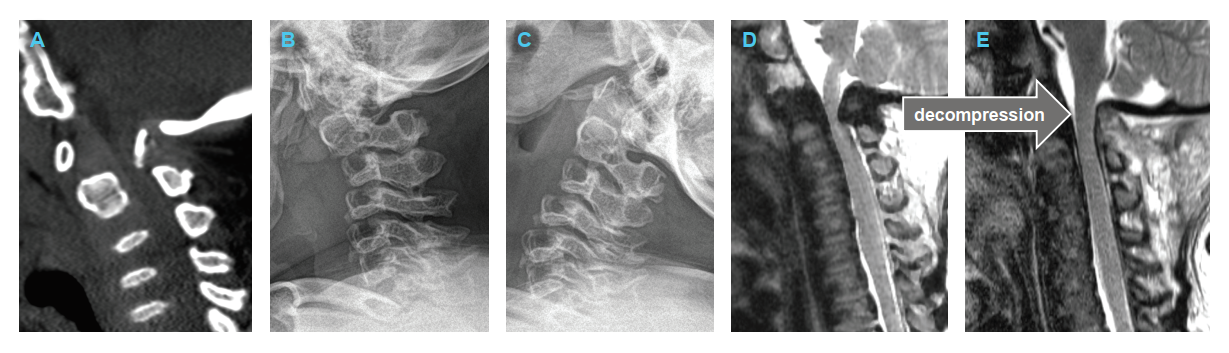


**Figure 3**. Radiographic findings of Patient 10 (severe): **A** odontoid hypoplasia, 15 years old; **B** and **C** atlantoaxial instability; **D** compressive myelopathy, 15.1 years old; **E** after C1 decompression.


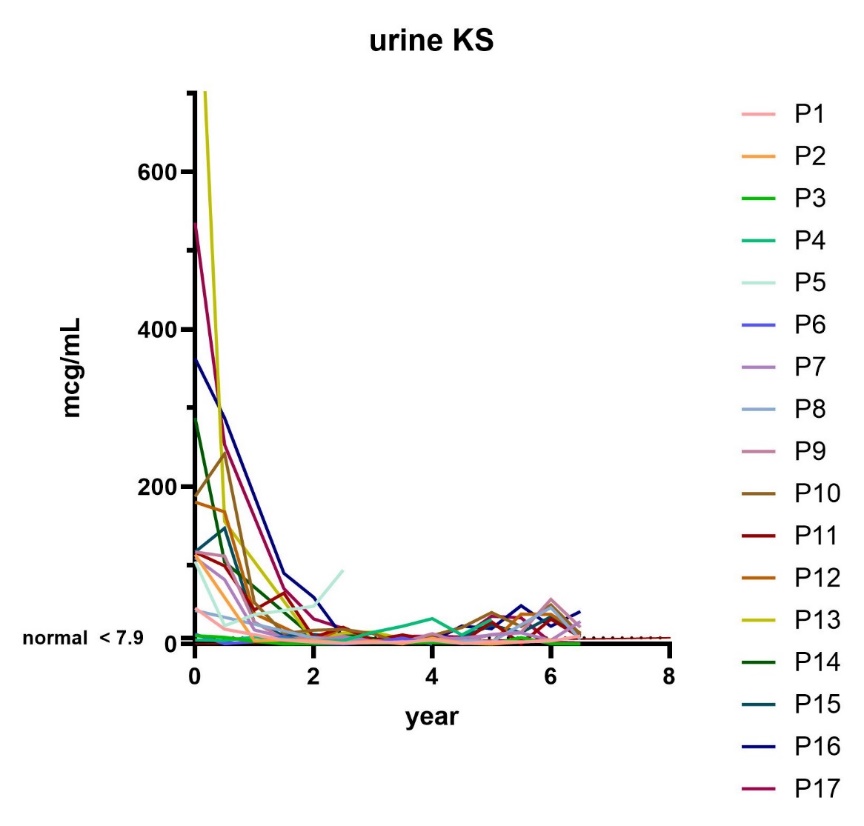


**Figure 4**. Change in urine keratan sulfate levels from pre-treatment baseline.


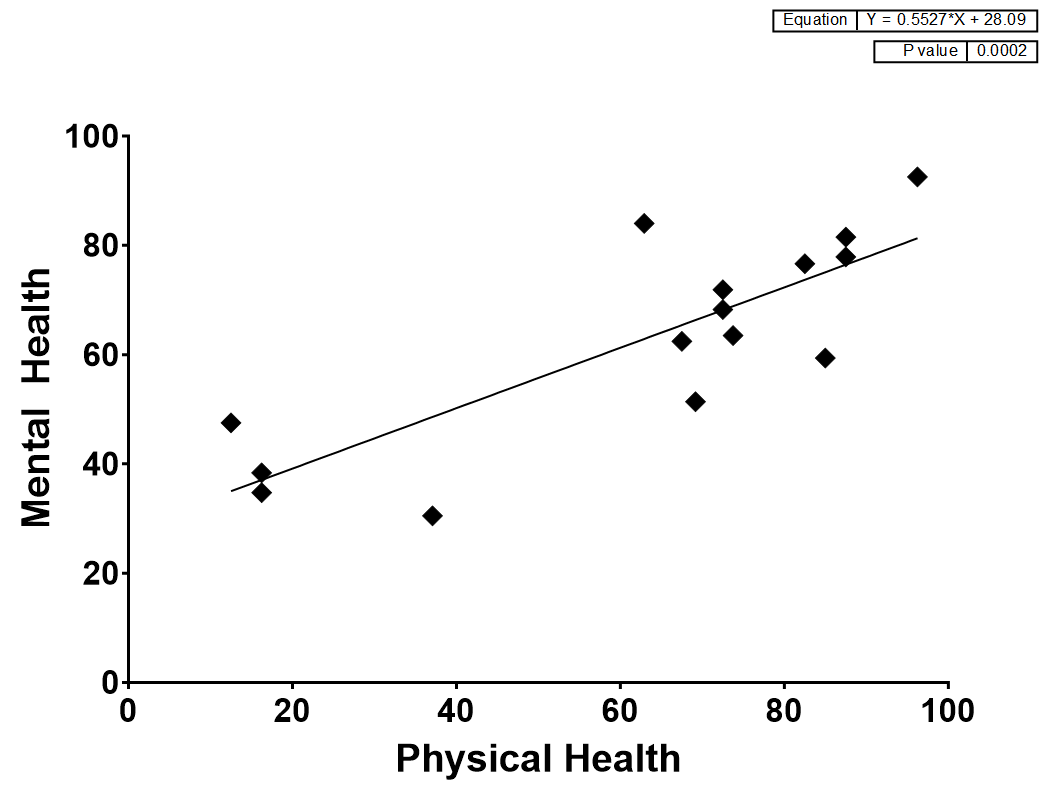


**Figure 5.** Correlation between physical health and mental health scores (Quality of Life Short Form-36).
